# MRKAd5 HIV-1 Gag/Pol/Nef Vaccine-Induced T-Cell Responses Inadequately Predict Distance of Breakthrough HIV-1 Sequences to the Vaccine or Viral Load

**DOI:** 10.1371/journal.pone.0043396

**Published:** 2012-08-27

**Authors:** Holly Janes, Nicole Frahm, Allan DeCamp, Morgane Rolland, Erin Gabriel, Julian Wolfson, Tomer Hertz, Esper Kallas, Paul Goepfert, David P. Friedrich, Lawrence Corey, James I. Mullins, M. Juliana McElrath, Peter Gilbert

**Affiliations:** 1 Division of Vaccines and Infectious Diseases, Fred Hutchinson Cancer Research Center, Seattle, Washington, United States of America; 2 Department of Biostatistics, University of Washington, Seattle, Washington, United States of America; 3 Department of Global Health, University of Washington, Seattle, Washington, United States of America; 4 United States Military HIV Research Program (MHRP), Rockville, Maryland, United States of America; 5 Division of Biostatistics, University of Minnesota, Minneapolis, Minnesota, United States of America; 6 Division of Clinical Immunology and Allergy, University of Sao Paulo, São Paulo, São Paulo, Brazil; 7 Department of Medicine, University of Alabama at Birmingham, Birmingham, Alabama, United States of America; 8 Department of Medicine, University of Washington, Seattle, Washington, United States of America; 9 Department of Microbiology, University of Washington, Seattle, Washington, United States of America; University of Cape Town, South Africa

## Abstract

**Background:**

The sieve analysis for the Step trial found evidence that breakthrough HIV-1 sequences for MRKAd5/HIV-1 Gag/Pol/Nef vaccine recipients were more divergent from the vaccine insert than placebo sequences in regions with predicted epitopes. We linked the viral sequence data with immune response and acute viral load data to explore mechanisms for and consequences of the observed sieve effect.

**Methods:**

Ninety-one male participants (37 placebo and 54 vaccine recipients) were included; viral sequences were obtained at the time of HIV-1 diagnosis. T-cell responses were measured 4 weeks post-second vaccination and at the first or second week post-diagnosis. Acute viral load was obtained at RNA-positive and antibody-negative visits.

**Findings:**

Vaccine recipients had a greater magnitude of post-infection CD8+ T cell response than placebo recipients (median 1.68% vs 1.18%; p = 0·04) and greater breadth of post-infection response (median 4.5 vs 2; p = 0·06). Viral sequences for vaccine recipients were marginally more divergent from the insert than placebo sequences in regions of Nef targeted by pre-infection immune responses (p = 0·04; Pol p = 0·13; Gag p = 0·89). Magnitude and breadth of pre-infection responses did not correlate with distance of the viral sequence to the insert (p>0·50). Acute log viral load trended lower in vaccine versus placebo recipients (estimated mean 4·7 vs 5·1) but the difference was not significant (p = 0·27). Neither was acute viral load associated with distance of the viral sequence to the insert (p>0·30).

**Interpretation:**

Despite evidence of anamnestic responses, the sieve effect was not well explained by available measures of T-cell immunogenicity. Sequence divergence from the vaccine was not significantly associated with acute viral load. While point estimates suggested weak vaccine suppression of viral load, the result was not significant and more viral load data would be needed to detect suppression.

## Introduction

The Step trial evaluated the efficacy of the Merck Adenovirus 5 (MRKAd5) Gag/Pol/Nef vaccine to prevent HIV-1 acquisition and reduce viral load. Three thousand high-risk HIV-1 negative individuals at 34 sites in North America, the Caribbean, South America, and Australia were randomized to vaccine or placebo. Immunizations were halted in September 2007 based on early evidence that the vaccine was ineffective at reducing HIV acquisition or viral load setpoint [1].

Rolland *et al.* [2] compared breakthrough HIV-1 sequences for male infected vaccine and placebo recipients to the vaccine insert sequence. They found greater protein distances to the insert sequence for vaccine recipients than for placebo recipients when restricting the analysis to regions with predicted T-cell epitopes. Importantly, this sieve effect was specific to the HIV proteins used in the vaccine, and was not found in other proteins. Thus, while the vaccine did not protect against infection, it did impact founding virus populations. Subsequent studies from the RV144 trial have suggested that reduction in acquisition of HIV-1 is associated with immune responses to envelope, a gene not included in the MRKAd5 vaccine [3].

This paper explores immunological and virological factors that may further elucidate the findings of Rolland *et al.* [2]. Specifically, we assessed whether pre- or post-infection T-cell responses were responsible for the viral sequence changes by correlating T-cell immunogenicity and viral sequence data. In addition, we evaluated whether the sieve effect on infecting viruses was associated with acute viral load. Our results indicate that while anamnestic responses were generated, the vaccine effect on viral sequences was largely not predicted by available measures of T-cell function. Neither was sequence divergence from the vaccine insert found to be significantly associated with viral load. There was some suggestion that the vaccine had a weak and transient impact on viral load, although the result was nonsignificant and future studies with more subjects with acute viral load data would be needed to confirm the effect.

## Methods

### Ethics Statement

The protocol was approved by the ethics review committee at every study site. These committees are: Emory University IRB # 2 and 3; Asociacion Civil Impacta Salud y Educacion IRB #1; University of Alabama at Birmingham IRB #1 and 2; Brigham and Women’s Hosp IRB #1 and 2; Massachusetts General Hospital IRB #1, 2 and 3; New York Blood Center, Inc.; Comite Institucional de Bioetica de Via Libre; University of Illinois at Chicago IRB #1; Columbia University Medical Center IRB #1, 2 and 3; Colorado Multiple Institutional Review Board; New York University School of Medicine IRB; Fenway Community Health IRB #1; Western IRB; Instituto Dermatologico y Cirugia de Piel IRB #1; CONABI - Biomedical Public Health; Asociacion Civil Impacta Salud Y Educacion IRB #1; Jamaica Ministry of Health IRB #1; University of the West Indies IRB #1; AIDS Research Alliance Institutional Review Board; Centre hospitalier de l’Universite de Montreal; North Jersey Community Research Initiative IRB; University of Pennsylvania IRB #1–5; Children’s Hospital of Philadelphia IRB #1 and 2; Haitian Group for the Study of Kaposi’s Sarcoma and Opportunistic Infections (GHESKIO) Center IRB #1; Joan and Sanford I Weill Medical College Cornell University IRB #2; Hospital Universitario Clementino Fraga Filho IRB #1; University of Rochester IRB #1; San Francisco General Hospital (SFGH) Committee IRB #2; Independent Review Consulting, Inc. (IRC) IRB #1; St. Vincent’s Hospital Human Research and Ethics Committee IRB #1; Merck Institutional Review Board #1; Providence Health Care IRB #1. All participants provided written informed consent.

### Population

The analysis included all 88 male subjects who were HIV uninfected at entry and diagnosed with HIV infection before unblinding on October 17, 2007, in addition to three males diagnosed within two months after unblinding but likely infected earlier (37 placebo and 54 vaccine recipients in total). Seventy (78%) were fully vaccinated, 20 (22%) had two vaccinations, and one (1%) had a single vaccination. One female was infected before study unblinding and was excluded from additional analysis. Figure S1 contains a diagram showing the various datasets that are the subject of this analysis.

### Subject Characteristics

The following baseline participant characteristics were included in certain analyses: race (White vs Non-white); age (≤ vs >30 years); self-reported circumcision status; HSV-2 serostatus; and HLA class I type (Protective: expressing HLA-B*57, B*5801 or B*27 in at least one allele; Unfavorable: expressing B*3502, *3503, *3504, or *5301 in at least one allele or homozygous in at least one locus; or Neutral: remaining subjects). Two North American subjects missing circumcision status were assumed to be circumcised, as 78% of other North American subjects in this study were circumcised. One subject missing HLA type and another missing HSV-2 serostatus were excluded from analyses involving these variables.

### Pre-infection Immunogenicity Data

Pre-infection T-cell responses were measured by a validated interferon-gamma (IFNγ) ELISpot assay [4] with Mabtech kits (Stockholm, Sweden) and vaccine-insert-matched peptides (Synpep, Dublin, CA) at 90% purity, using PBMC samples obtained four weeks post-second vaccination. Samples were stimulated with pools of peptides 15 amino acids in length and overlapping in sequence by 11 amino acids at a final concentration of 1 µg/ml per peptide. Gag, Pol, and Nef pools contained 122, 210, and 51 peptides, respectively. The magnitude of the response was characterized by the number of spot-forming cells per million PBMC [5] and positive response criteria were previously published [4]. Data were available for 46 of the 54 vaccine recipients; eight were excluded due to HIV-1 infection at the time of analysis.

Responses were mapped to individual 15-mers for 37 of the 46 vaccine recipients as previously described using a final concentration of 2 µg/ml per peptide [4]. Data were missing for seven due to insufficient numbers of cells and two due to high background. The breadth of the response was measured by the number of reactive 15-mers, with two overlapping 15-mers counted as a single response.

### Viral Sequence Data

Plasma for viral sequencing was obtained from the first available HIV PCR positive visit (or one month later for one individual) for 67 of the 91 subjects, 27 to 741 days after enrollment (median = 231 days). Sequence data were missing for 13 subjects because specimens were not available and for 11 subjects because HIV-1 could not be amplified by PCR.

Rolland *et al.* [2] found significant differences between vaccine and placebo sequences in regions that were predicted to be T-cell epitopes based on a subject’s HLA alleles. They defined the “predicted CTL epitope” distance between a breakthrough sequence and the MRKAd5 insert as the HIV-specific evolutionary (PAM) distance [6] in peptides predicted to be epitopes in both sequences, averaged over a subject’s breakthrough sequences. The “breakthrough K-mers distance” is the percentage of predicted epitopes in the insert sequence that mismatch at least one breakthrough sequence. Two versions of each distance were computed using different algorithms for predicting epitopes, Epipred [7] or NetMHC [8]. Rolland *et al.* [2] also performed a “signature analysis” to identify amino acid sites and K-mers at which the rate of amino acid/peptide mismatch to the insert differed between breakthrough sequences in vaccine and placebo groups. Ten signature sites (Gag-84, 211, 403, and 465; Pol-541 and 721; and Nef-64a, 92, 116, and 173) and 14 signature 9-mers (beginning at Gag-204, Gag-372, two contiguous 9-mers beginning at Gag-382, six contiguous 9-mers beginning at Gag-398, and four contiguous 9-mers beginning at Nef-121) were detected and were assessed in this analysis.

### Viral Load Data

Acute viral load was defined as a plasma viral load measured from an HIV-RNA-positive, ELISA-negative, and Western Blot borderline or negative sample. The HIV diagnostic and viral load assays are described in Buchbinder *et al.* [1]. Twenty-seven subjects (14 placebo and 13 vaccine recipients) had an acute viral load measurement available for analysis; for the remaining 64 subjects no samples were collected pre-seroconversion. Of the 19 subjects who had both acute viral load and viral sequence data, 18 had data from the same visit and one had sequence data 28 days after the acute viral load measurement.

### Post-infection Immunogenicity Data

Post-infection T-cell responses were measured at week one (n = 7) or two (n = 72) post-infection diagnosis by intracellular cytokine staining (ICS) using pools of PTE-G peptides (Biosynthesis, Lewisville, TX [9]) at a final concentration of 1 µg/ml per peptide as described previously [5], [10]. Gag, Pol, and Nef pools contained 160, 160, and 127 peptides, respectively. Post-infection responses were available for 79 participants; the remaining 12 had insufficient cells for testing. The primary measure of immunogenicity was the percent of CD4+ or CD8+ T-cells expressing interleukin-2 (IL2) and/or IFNγ. Positive responses were determined using previously published ICS positivity criteria [10]. Positive peptide pool responses were mapped to individual 15-mers using IFNγ ELISpot as described previously [4]. Breadth was defined by the number of reactive 15-mers, with two overlapping 15-mers counted as a single response. Data were available for 36 subjects and were missing for one due to low cell viability and 54 due to sample availability. To evaluate preservation of pre-infection responses, for 23 vaccine recipients, post-infection responses to individual vaccine-insert-matched 15-mers which generated pre-infection responses were also assessed using IFNγ ELISpot. Insufficient cells were available for the remaining 23 vaccine recipients with pre-infection T-cell data.

### Statistical Methods

The methods are described adjacent to each analysis result for readability. All p-values are two-sided.

## Results

### Vaccine-induced Anamnestic Responses

**Figure 1:**
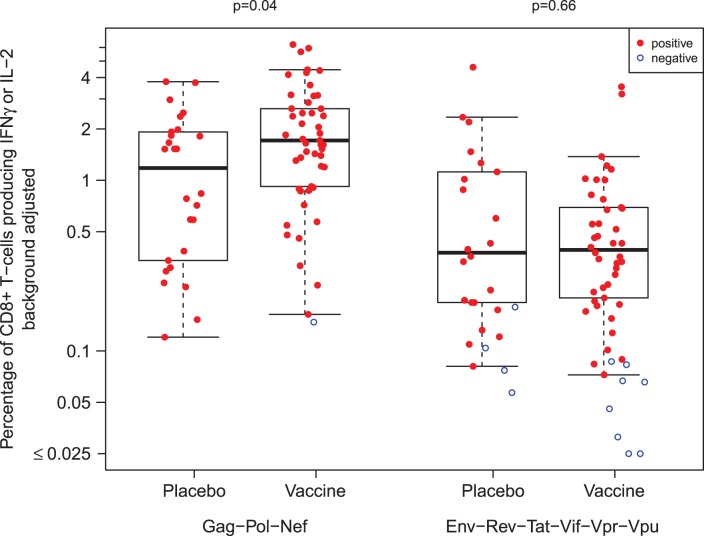
Post-Infection Magnitude of CD8+ T-Cell Response. Magnitude of the post-infection CD8+ T-cell response measured by ICS, as quantified by the percentage of CD8+ T-cells producing IFN or IL-2 when stimulated with the vaccine-insert-matched peptide pools (Gag, Pol, and Nef) and other non-vaccine-insert peptide pools, for vaccine and placebo groups. Positive responses are indicated using closed red circles and negative responses using open blue circles. The p-values refer to tests comparing response magnitudes between the vaccine and placebo positive responders.

One potential mechanism for the observed sieve effect is vaccine-induced anamnestic responses. We compared the magnitude of the post-infection ICS response between vaccine and placebo positive responders using a Wilcoxon rank sum test (n = 79 subjects; Figure 1). The magnitude of the CD8+ T-cell response was found to be greater in vaccine versus placebo recipients (p = 0·04). The median Gag-Pol-Nef magnitude was 1.68% in vaccine recipients (SD = 1.50%) versus 1.18% in placebo recipients (SD = 1.09%). CD4+T-cell responses did not differ between treatment groups (p = 0·54; data not shown). Non-insert responses for CD4+ and CD8+ T-cells were similar between the groups (p>0·50).

**Figure 2:**
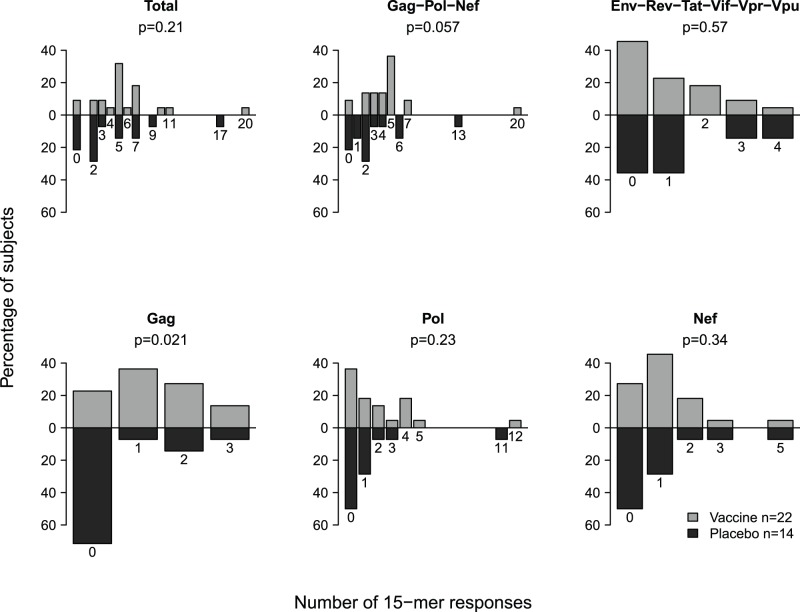
Post-Infection Breadth of T-Cell Response. Breath of the post-infection T-cell response as measured by IFNγ ELISpot, as quantified by the number of reactive 15-mers, for the vaccine (grey) and placebo (black) groups. The distribution of breadth is shown for all proteins in aggregate; for Gag, Pol, and Nef combined; for other non-insert proteins; and for Gag, Pol, and Nef individually. The p-values refer to tests comparing breadth between vaccine and placebo groups.

Epitope breadth was also compared between treatment groups using a Wilcoxon rank sum test (n = 36 subjects; Figure 2). Median Gag-Pol-Nef breadth was 4.5 vs 2 in vaccine vs placebo recipients. Breadth of the Gag response was greater in vaccine versus placebo recipients (p = 0·02) but no difference was detected for Pol or Nef (p = 0·23 and 0·34, respectively).

**Figure 3:**
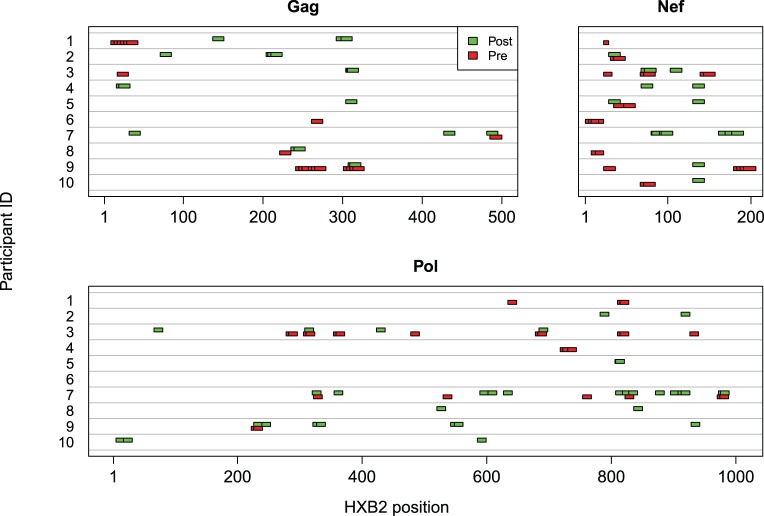
Comparison of Pre- and Post-Infection T-Cell Responses. Pre- and post-infection T-cell responses to individual 15-mers in Gag, Pol, and Nef as measured by IFNγ ELISpot. Each row represents a different subject. Pre-infection responses were measured using vaccine-matched peptides and post-infection responses were measured using PTE-G peptides.

HIV infection induced a number of immune responses that were undetected pre-infection (Figure 3). Based on the 23 vaccine recipients whose pre-infection responses were tested post-infection, on average 63·0% of pre-infection responses to Gag-Pol-Nef were preserved post-infection. Additional analyses of anamnestic responses are reported in Materials S1 Section 1 (see Table S1; Figures S2, S3, S4).

### Integrated Analysis of Viral Sequences and T-cell Responses

#### T-cell response based sieve analysis

Vaccine-induced T-cell selection pressure would be expected to lead to viral sequence differences in some fraction of reactive epitopes due to immune escape. Specifically, within the measured pre-infection reactive epitopes, we would expect an unusually high rate of mismatches in vaccine recipient founder sequence epitopes compared to the corresponding insert epitopes. To address this hypothesis, for each vaccine recipient we estimated the percentage of mismatches as the observed rate at which any of his founder sequences mismatched the insert residue, across all sites within his reactive 15-mers. These percentages were averaged across the vaccine recipients with at least one positive response, for each protein region. The 27 placebo subjects with viral sequences were bootstrapped to generate a null distribution and compute a p-value.

Among the 32 vaccine recipients with sequence and pre-infection T-cell response data, 13, 16, and 16 had an ELISpot response to at least one 15-mer in Gag, Pol, and Nef, respectively. There were more mismatches than expected by chance in Nef (p = 0·04), a non-significant trend for Pol (p = 0·13), and no significant evidence for Gag (p = 0·89). The average percent mismatch for vaccine recipients, with the median for the null distribution shown in parentheses, was calculated as 26.1% (23.3%) for Nef, 6.7% (5.5%) for Pol, and 8.5% (8.3%) for Gag. Results were similar considering only the most immunodominant [11] or the most conserved 15-mer for each vaccine recipient (see Materials S1 Section 2 and Figure S5).

#### Association between pre-infection T-cell responses and epitope-based distance measures

If vaccine-induced T-cell responses led to immune escape, vaccine recipients with greater pre-infection immune responses would be expected to have viral epitope sequences with greater distance to the vaccine insert. We examined associations between the magnitude and breadth of the pre-infection IFNγ ELISpot response (within and across proteins) and each epitope-based distance measure in the 32 vaccine recipients with pre-infection T-cell response and sequence data. Each participant’s majority consensus founder sequence was used; this is the most common amino acid at each individual site. In cases of multiple founders, the consensus of the largest founding population was used. Linear regression was used to compare mean protein distance by breadth, categorized as: 0 vs. 1–2 vs. >2 reactive 15-mers; and by log_10_ magnitude of response to the peptide pools. Seven subjects (22%) had 0 breadth, 6 (19%) had breadth 1–2, and 19 (59%) had breadth >2. There were no significant associations between the pre-infection magnitude or breadth of the vaccine-induced response to a protein and protein distance in the same or different region (p>0·7 for all pairs of distance and breadth).

#### Locations of signature sites in relation to pre-infection T-cell responses

**Figure 4:**
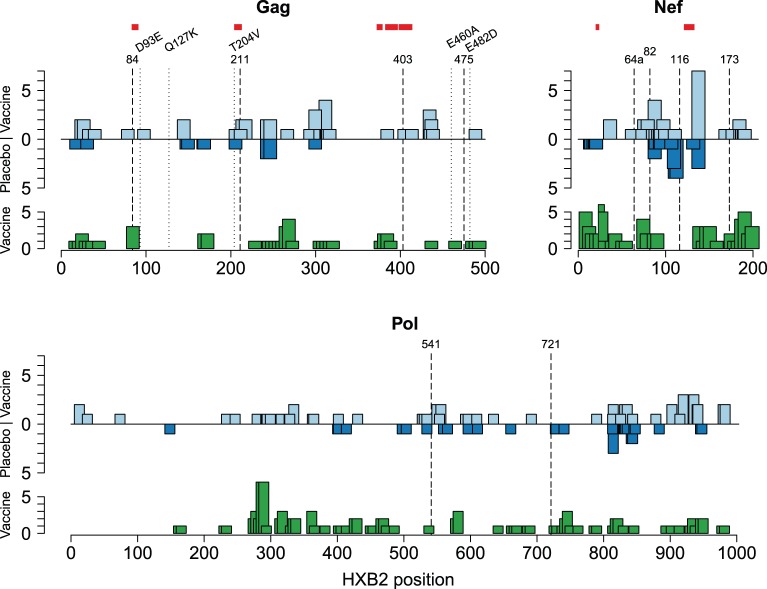
Signature Sites in Relation to T-Cell Responses. Locations of the signature sites and pre- and post-infection T-cell responses for Gag, Nef, and Pol. For each protein, the top graph represents the location of post-infection IFNγ ELISpot responses detected in 19 vaccine and 11 placebo recipients (light and medium blue columns, respectively), signature K-mers (red horizontal bars), amino acid signature sites (dashed vertical lines), and sites where an insert-mismatch was found to be associated with viral load in the vaccine group alone (dotted vertical lines; Materials S1 Section 5). The bottom graph corresponds to pre-infection IFNγ ELISpot responses detected in 27 vaccine recipients (green columns). The region covered by each responsive peptide is indicated by the box width, and the number of subjects reacting to that peptide is indicated by the box height.

IFNγ ELISpot detected pre-infection responses to four of the ten signature sites identified by Rolland *et al.*: [2] Gag-84, Pol-541, Nef-82, and Nef-173 (Figure 4). Three subjects recognized the Gag-84 (T/V) signature. One HLA-A*02 individual recognized the A02-restricted epitope SLYNTVA**T**L [12] and two HLA-A*11 individuals recognized the A11-restricted epitope **T**LYCVHQK [13]. For the Nef-82 (K/not-K) signature, two HLA-A*11 individuals recognized the A11-restricted epitope AVDLSHFLK, just downstream of the signature site (Nef 84–92). One HLA-A*03 subject recognized the A03-restricted epitope QVPLRPMTY**K**, and one subject recognized the 15-mer but the HLA-restricted epitope was unknown. There were no known epitopes in the 15-mers covering the Pol-541 or Nef-173 signature sites.

### Acute Viral Load Analysis

**Figure 5:**
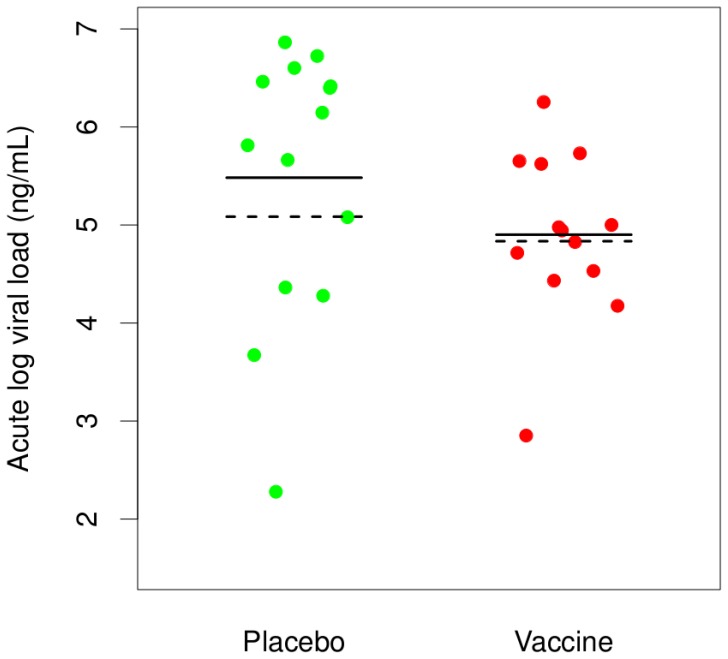
Acute Log_10_ Viral Load. The distribution of acute log_10_ viral load values in vaccine and placebo groups. Solid lines correspond to observed means and dashed lines correspond to means estimated using the multiple imputation approach.

We evaluated evidence of a vaccine-induced reduction in acute viral load by comparing the distribution of log_10_ acute viral load between treatment groups using data from all 91 subjects. Sixty-four missing viral load values were multiply imputed using an indicator of unfavorable HLA type, study week of HIV diagnosis, race, age, and circumcision status; and results were combined across 20 imputed datasets using standard multiple imputation rules [14]. The estimated mean log acute viral load was lower in vaccine versus placebo recipients (4·7 vs 5·1; Figure 5) but the difference was not significant (p = 0·27 Wilcoxon rank sum; p = 0·21 t-test). Analyses using alternative missing data methods produced qualitatively similar results (Materials S1 Section 4). A trend towards reduced acute viral load in the vaccine group was also seen after stratifying the data by HLA-progression-type (Figure S6).

### Integrated Analysis of Viral Sequences and Acute Viral Load

We assessed whether there was an association between breakthrough sequences and acute viral load using the 67 subjects with viral sequence data. Majority consensus founder sequences were used and missing viral load values were imputed as described above. Each epitope-based distance measures was associated with acute log viral load using a linear model with treatment assignment, summary distance measure, and an interaction between treatment and distance as predictors. No associations were detected in vaccine or placebo groups in any individual protein or aggregating across Gag, Pol, and Nef (p>0·3 for all tests), nor was there evidence of a significant difference in association between treatment groups (p>0·3 for all interaction tests; Figures S7, S8, S9, S10, S11).

Each individual amino acid site was also examined. A Wilcoxon rank sum test was used to evaluate differences in acute viral load between vaccine and placebo recipients with an insert-matched residue (n = 753 sites assessed). Where nominal p-values were less than 0·05, q-values measuring the positive false-discovery rate were calculated [15]–[17]. We found no individual amino acid sites in Gag, Pol, or Nef with treatment differences (p-values ranging from 0·1 to 1·0). Five sites in Gag were found to be associated with viral load in the vaccine group alone (Materials S1 Section 5).

## Discussion

This study found clear evidence of vaccine-induced anamnestic responses for CD8+ but not CD4+ T-cells. However the T-cell response data only partially explained the vaccine-induced sequence changes identified by Rolland *et al.* [2]. Pre-infection T-cell responses did not predict divergence of the breakthrough sequences from the vaccine insert, yet there was some evidence of differences between vaccine and placebo breakthrough sequences in regions with observed pre-infection T-cell responses. This evidence was concentrated in Nef which has been shown to be highly immunodominant during acute infection [18].

Analyses of the T-cell data were challenged by methodological and data availability issues. T-cell responses were sparse such that few epitopes were recognized by multiple individuals; hence we had limited power to detect a sieve effect in immunofocused regions and to correlate the T-cell responses and sequence data. The T-cell response-based sieve analysis also had limited power since only a fraction of epitopes may experience selection pressure due to an immune response. We were limited by sample availability and minimal overlap between the viral sequence and immunogenicity data as well. In addition, methodological improvements, such as refinement of epitope-mapping algorithms and novel assays, are needed to more directly measure vaccine-induced T-cell responses. Finally, the immune responses we measured in the blood do not necessarily reflect responses in the mucosal tissue which play a key role in early infection.

The vaccine did not significantly reduce acute log viral load, although the point estimate of a 0.4 log_10_ reduction suggests the possibility of weak suppression that would need confirmation in a study with more acute viral load data. Separate analyses of viral load data in Step from HIV diagnosis to 4 years post-infection (median follow-up two years post-infection) demonstrated that the vaccine did not reduce post-acute viral load [19]. Therefore any impact of the vaccine on viral load was early and transient.

We tested the following hypotheses regarding the impact of the viral sequence changes on acute viral load. If vaccine-induced changes in breakthrough sequences led to decreased viral fitness in terms of viral load, we would expect higher epitope-based distances to be associated with lower acute viral load, differentially so in the vaccine group. There are a few examples in the literature of CTL escape being associated with reduced viral fitness [20]–[22], although the SLYNTVATL epitope that encompasses the Gag-84 signature site is well known to escape without fitness cost to the virus. Alternatively, if vaccine-induced T-cells recognized certain epitopes and were therefore able to suppress viral replication, we would expect lower epitope-based distances to be associated with lower acute viral load among vaccine recipients, but no association in placebo recipients. Our analyses linking acute viral load with viral sequence data were designed to be sensitive to either hypothesis. We found no detectable evidence of a fitness cost as measured by acute viral load, or of reduced viral replication due to vaccine-induced epitope recognition.

The viral load analyses were also limited by low power. Acute viral load was missing for 70% of subjects, largely due to the Step study’s six-monthly HIV testing from Week 30 on (four weeks after the last vaccination). This highlights the importance of more frequent HIV testing in efficacy trials. Had HIV testing been more frequent, we would likely have been adequately powered to detect a vaccine effect on acute viral load of the size estimated here, a reduction of 0·4 log.

Sieve analysis plays a key role in the assessment of immune correlates. Specifically, it can be useful for identifying the specific immune responses responsible for any observed vaccine efficacy. This study emphasizes the need for immune correlates assessments to be based on assays that are epitope-specific and that are rigorously validated and have high reproducibility.

Despite detecting vaccine-induced anamnestic responses, this study found that the observed T-cell responses did not adequately explain the vaccine effect on founding virus populations identified by Rolland *et al.* [2]. Importantly, this implies that the measures of T-cell response that were employed did not adequately explain some immune functions that were nonetheless able to put pressure on the virus. Possible explanations include a lack of sensitivity of our immune assays or use of inappropriate assays. Neither was there evidence of a consequence of the viral sequence changes in terms of acute viral load. Nevertheless, the trend toward modest viral load suppression in vaccine recipients suggests a hypothesis that vaccines with improved CD8+ T cell responses may be able to exert stronger pressure with greater fitness cost and improved viral load suppression.

## Supporting Information

Figure S1 Datasets Included in the Analysis and Associated Scientific Questions.
(EPS)

Figure S2 Post-Infection Depth of T-Cell Response. Depth of the post-infection response for vaccine (V) and placebo (P) recipients. Depth at an amino acid site is defined as the number of simultaneously elicited variant PTE-G peptides that cover the site (using HXB2 numbering). The distribution of depth is shown for Gag, Pol, and Nef combined; for Gag, Pol, and Nef individually, and for all non-insert proteins. The p-values refer to tests comparing depth between vaccine and placebo groups.
(EPS)

Figure S3 Post-Infection Responses to Peptides Covering Signature Sites. The distribution of the number of positive 15-mers covering the signature sites, for vaccine and placebo groups.
(EPS)

Figure S4 Difference in Magnitude of Positive T-Cell Response Post- vs Pre-Infection. The difference in the log_10_ magnitude T-cell response post- minus pre-infection among the 23 vaccine recipients whose positive responses pre-infection were tested post-infection. Only positive post-infection responses are shown. The p-values refer to tests comparing the mean differences to zero.
(EPS)

Figure S5 Results of T-Cell Response Based Sieve Analysis: The Null Distribution and Observed Average Mismatch Rate. Histograms show the bootstrap null distribution of the average mismatch rate and the vertical lines indicate the observed average mismatch rate. The two-sided p-value is the fraction of the bootstrap statistics that are more extreme than the observed statistic. The top row shows these figures for Gag, Pol, and Nef. The second row restricts to “immunodominant” responses and the third row to “conserved” responses.
(EPS)

Figure S6 Acute Log_10_ Viral Load by HLA Group. The distribution of acute log_10_ viral load values in vaccine and placebo recipients by HLA group. HLA groups are defined as protective (B27, B57, B5801), unfavorable (B*3502, *3503, *3504, B53, or homozygous in at least one locus), and neutral haplotypes (all others). Solid lines correspond to observed means and dashed lines correspond to means estimated using the multiple imputation approach.
(EPS)

Figure S7 Association Between Acute Log_10_ Viral Load and Each Summary Distance Measure (Gag-Pol-Nef Total). The predicted CTL epitope distance between a breakthrough sequence and the MRKAd5 insert is the HIV-specific evolutionary (PAM) distance in peptides predicted to be epitopes in both sequences, averaged over a subject’s breakthrough sequences. The breakthrough K-mers distance is the percentage of predicted epitopes in the insert sequence that mismatch at least one breakthrough sequence. Epitopes were predicted using NetMHC and Epipred. Missing viral load values were multiply imputed and represent averages across 20 datasets; solid points are observed values and open points are averages across 20 imputations. The Pearson correlation (r) is shown for vaccine (red circles; dashed line) and placebo (green squares; solid line) groups. Fitted lines from linear regression models are overlaid.
(EPS)

Figure S8 Association Between Acute Log_10_ Viral Load and Breakthrough K-mers NetMHC Distance. The breakthrough K-mers distance is the percentage of NetMHC-predicted epitopes in the insert sequence that mismatch at least one breakthrough sequence. Missing viral load values were multiply imputed and represent averages across 20 datasets; solid points are observed values and open points are averages across 20 imputations. The Pearson correlation (r) is shown for vaccine (red circles; dashed line) and placebo (green squares; solid line) groups. Fitted lines from linear regression models are overlaid.
(EPS)

Figure S9 Association Between Acute Log_10_ Viral Load and Breakthrough K-mers Epipred Distance. The breakthrough K-mers distance is the percentage of Epipred-predicted epitopes in the insert sequence that mismatch at least one breakthrough sequence. Missing viral load values were multiply imputed and represent averages across 20 datasets; solid points are observed values and open points are averages across 20 imputations. The Pearson correlation (r) is shown for vaccine (red circles; dashed line) and placebo (green squares; solid line) groups. Fitted lines from linear regression models are overlaid.
(EPS)

Figure S10 Association Between Acute Log_10_ Viral Load and Predicted CTL Epitope NetMHC Distance. The predicted CTL epitope distance between a breakthrough sequence and the MRKAd5 insert is the HIV-specific evolutionary (PAM) distance in peptides predicted to be epitopes in both sequences (based on NetMHC), averaged over a subject’s breakthrough sequences. Missing viral load values were multiply imputed and represent averages across 20 datasets; solid points are observed values and open points are averages across 20 imputations. The Pearson correlation (r) is shown for vaccine (red circles; dashed line) and placebo (green squares; solid line) groups. Fitted lines from linear regression models are overlaid.
(EPS)

Figure S11 Association Between Acute Log_10_ Viral Load and Predicted CTL Epitope Epipred Distance. The predicted CTL epitope distance between a breakthrough sequence and the MRKAd5 insert is the HIV-specific evolutionary (PAM) distance in peptides predicted to be epitopes in both sequences (based on Epipred), averaged over a subject’s breakthrough sequences. Missing viral load values were multiply imputed and represent averages across 20 datasets; solid points are observed values and open points are averages across 20 imputations. The Pearson correlation (r) is shown for vaccine (red circles; dashed line) and placebo (green squares; solid line) groups. Fitted lines from linear regression models are overlaid.
(EPS)

Table S1 List of the 32 peptides covering the signature sites that were assessed using IFNγ ELISpot.
(DOCX)

Materials S1 (DOCX)

## References

[1] Buchbinder SP, Mehrotra DV, Duerr A, Fitzgerald DW, Mogg R, et al (2008) Efficacy assessment of a cell-mediated immunity HIV-1 vaccine (the Step Study): a double-blind, randomised, placebo-controlled, test-of-concept trial. Lancet 372: 1881–93. 19012954 10.1016/S0140-6736(08)61591-3 PMC2721012

[2] Rolland M, Tovanabutra S, DeCamp AC, Frahm N, Gilbert PB, et al (2011) Genetic impact of vaccination on breakthrough HIV-1 sequences from the STEP trial. Nat Med. 17: 366–71. 10.1038/nm.2316 PMC3053571 21358627

[3] Haynes BF, Gilbert PB, McElrath MJ, Zolla-Pazner S, Tomaras GD, et al (2012) Immune-correlates analysis of an HIV-1 vaccine efficacy trial. N Engl J Med. 366(14): 1275–86. 10.1056/NEJMoa1113425 PMC3371689 22475592

[4] Frahm N, DeCamp AC, Friedrich DP, Carter DK, Defawe OD, et al.. (2012) Human adenovirus-specific T cells modulate HIV-specific T-cell responses to an Ad5-vectored HIV-1 vaccine. J. Clin Invest (in press). 10.1172/JCI60202 PMC3248307 22201684

[5] McElrath MJ, De Rosa SC, Moodie Z, Dubey S, Kierstead L, et al (2008) HIV-1 vaccine-induced immunity in the test-of-concept Step Study: A case-cohort analysis. Lancet 372: 1894–05. 19012957 10.1016/S0140-6736(08)61592-5 PMC2774110

[6] Nickle DC, Rolland M, Jensen MA, Pond SL, Deng W, et al (2007) Coping with viral diversity in HIV vaccine design. PLoS Comput Bio 3: e75. 17465674 10.1371/journal.pcbi.0030075 PMC1857809

[7] Heckerman D, Kadie C, Listgarten J (2007) Leveraging information across HLA alleles/supertypes improves epitope prediction. J Comput Biol 14: 736–46. 17691891 10.1089/cmb.2007.R013

[8] Buus S, Lauemoller SL, Worning P, Kesmir C, Frimurer T, et al (2003) Sensitive quantitative predictions of peptide-MHC binding by a ‘Query by Committee’ artificial neural network approach. Tissue Antigens 62: 378–84. 14617044 10.1034/j.1399-0039.2003.00112.x

[9] Li F, Malhotra U, Gilbert PB, Hawkins NR, Duerr AC, et al (2006) Peptide selection for human immunodeficiency virus type 1 CTL-based vaccine evaluation. Vaccine 24: 6893–04. 16890329 10.1016/j.vaccine.2006.06.009

[10] Horton H, Thomas EP, Stucky JA, Frank I, Moodie Z, et al (2007) Optimization and validation of an 8-color intracellular cytokine staining (ICS) assay to quantify antigen-specific T-cells induced by vaccination. J Immunol Methods 323: 39–54. 17451739 10.1016/j.jim.2007.03.002 PMC2683732

[11] Barouch DH, O’Brien KL, Simmons NL, King SL, Abbink P, et al (2010) Mosaic HIV-1 vaccines expand the breadth and depth of cellular immune responses in rhesus monkeys. Nat Med. 16: 319–23. 10.1038/nm.2089 PMC2834868 20173752

[12] Johnson RP, Trocha A, Yang L, Mazzara GP, Panicali DL, et al (1991) HIV-1 Gag-specific cytotoxic T lymphocytes recognize multiple highly conserved epitopes: Fine specificity of the Gag-specific response defined by using unstimulated peripheral blood mononuclear cells and cloned effectir cells. J Immunol 147: 1512–21. 1715361

[13] Harrer T, Harrer E, Barbosa P, Kaufmann F, Wagner R, et al (1998) Recognition of two overlapping CTL epitopes in HIV-1 p17 by CTL from a long-term nonprogressing HIV-1-infected individual. J Immunol 161: 4875–81. 9794421

[14] Rubin DB (1987) Multiple Imputation for Nonresponse in Surveys. Hoboken: John Wiley and Sons.

[15] Benjamini Y, Hochberg Y (1995) Controlling the false discovery rate: a practical and powerful approach to multiple testing. J R Stat Society Series B-Methodological 57: 289–300.

[16] Storey JD (2003) The positive false discovery rate: a Bayesian interpretation and the q-value. Ann Statistics 31: 2013–35.

[17] Storey J, Tibshirani R (2003) Statistical significance for genomewide association studies. PNAS 100: 9440–45. 12883005 10.1073/pnas.1530509100 PMC170937

[18] Licterfeld M, Yu XG, Cohen D, Addo MM, Malenfant J, et al (2004) HIV-1 Nef is preferentially recognized by CD8 T cells in primary HIV-1 infection despite a relatively high degree of genetic diversity. AIDS 18: 1383–92. 15199314 10.1097/01.aids.0000131329.51633.a3

[19] Fitzgerald DW, Janes H, Robertson M, Coombs R, Frank I, et al (2011) An Ad5-vectored HIV-1 vaccine elicits cell-mediated immunity but does not affect disease progression in HIV-1-infected male subjects: results from a randomized placebo-controlled trial (the Step study). J Infect Dis. 203: 765–72. 10.1093/infdis/jiq114 PMC3119328 21343146

[20] Martinez-Picado J, Prado JG, Fry EE, Pfafierott K, Leslie A, et al (2006) Fitness cost of escape mutations in p24 Gag in association with control of human immunodeficiency virus type 1. J Virol 80: 3617–23. 16537629 10.1128/JVI.80.7.3617-3623.2006 PMC1440414

[21] Schneidewind A, Brockman MA, Yang R, Adam RI, Li B, et al (2007) Escape from the dominant HLA-B27-restricted cytotoxic T-lymphocyte response in Gag is associated with a dramatic reduction in human immunodeficiency virus type 1 replication. J Virol 81: 12382–93. 17804494 10.1128/JVI.01543-07 PMC2169010

[22] Troyer RM, McNevin J, Liu Y, Zhang SC, Krizan RW, et al.. (2009) Variable fitness impact of HIV-1 escape mutations to cytotoxic T lymphocyte (CTL) response. PLoS Pathog (in press). 10.1371/journal.ppat.1000365 PMC2659432 19343217

