# Supplementary figures and images for: MRKAd5 HIV-1 Gag/Pol/Nef Vaccine-Induced T-Cell Responses Inadequately Predict Distance of Breakthrough HIV-1 Sequences to the Vaccine or Viral Load
Source: PLoS One. 2012 Aug 27;7(8):e43396. doi: 10.1371/journal.pone.0043396 (PMC3428369; doi:10.1371/journal.pone.0043396)

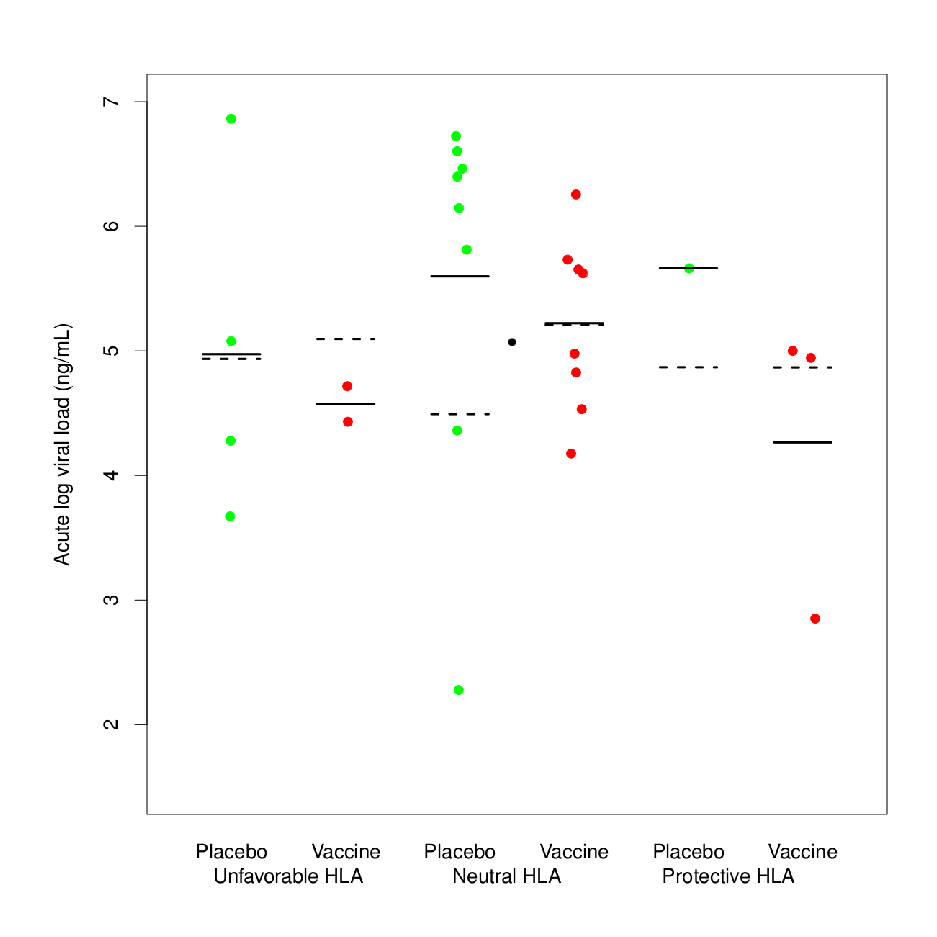

Supplement: Figure S6 — Acute Log10 Viral Load by HLA Group. The distribution of acute log10 viral load values in vaccine and placebo recipients by HLA group. HLA groups are defined as protective (B27, B57, B5801), unfavorable (B*3502, *3503, *3504, B53, or homozygous in at least one locus), and neutral haplotypes (all others). Solid lines correspond to observed means and dashed lines correspond to means estimated using the multiple imputation approach. (EPS) [file pone.0043396.s006.tif]

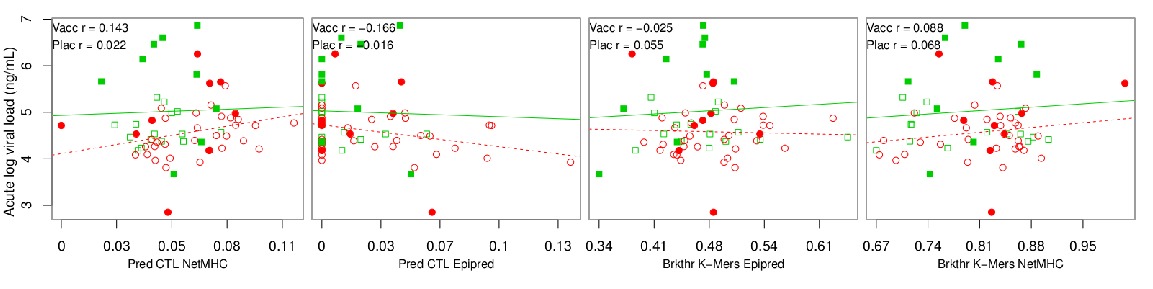

Supplement: Figure S7 — Association Between Acute Log10 Viral Load and Each Summary Distance Measure (Gag-Pol-Nef Total). The predicted CTL epitope distance between a breakthrough sequence and the MRKAd5 insert is the HIV-specific evolutionary (PAM) distance in peptides predicted to be epitopes in both sequences, averaged over a subject’s breakthrough sequences. The breakthrough K-mers distance is the percentage of predicted epitopes in the insert sequence that mismatch at least one breakthrough sequence. Epitopes were predicted using NetMHC and Epipred. Missing viral load values were multiply imputed and represent averages across 20 datasets; solid points are observed values and open points are averages across 20 imputations. The Pearson correlation (r) is shown for vaccine (red circles; dashed line) and placebo (green squares; solid line) groups. Fitted lines from linear regression models are overlaid. (EPS) [file pone.0043396.s007.tif]

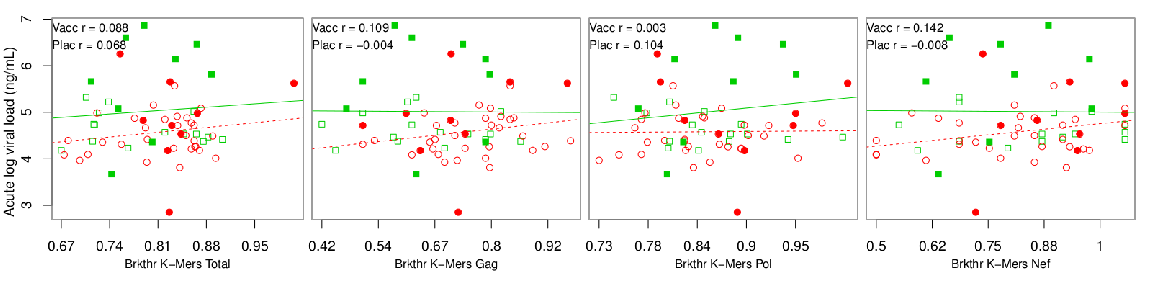

Supplement: Figure S8 — Association Between Acute Log10 Viral Load and Breakthrough K-mers NetMHC Distance. The breakthrough K-mers distance is the percentage of NetMHC-predicted epitopes in the insert sequence that mismatch at least one breakthrough sequence. Missing viral load values were multiply imputed and represent averages across 20 datasets; solid points are observed values and open points are averages across 20 imputations. The Pearson correlation (r) is shown for vaccine (red circles; dashed line) and placebo (green squares; solid line) groups. Fitted lines from linear regression models are overlaid. (EPS) [file pone.0043396.s008.tif]

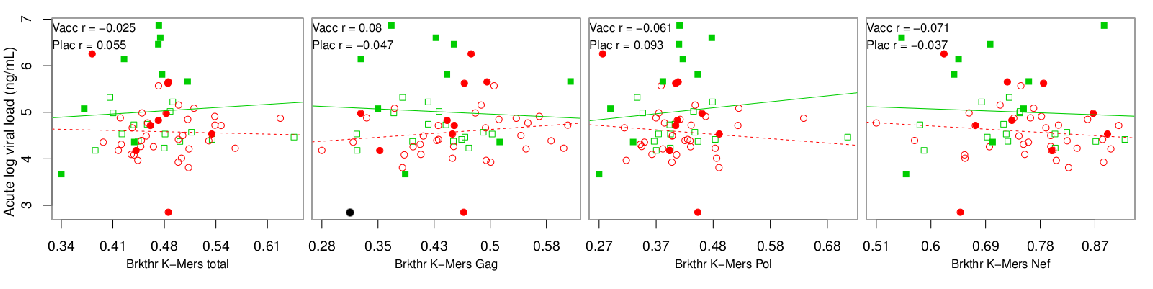

Supplement: Figure S9 — Association Between Acute Log10 Viral Load and Breakthrough K-mers Epipred Distance. The breakthrough K-mers distance is the percentage of Epipred-predicted epitopes in the insert sequence that mismatch at least one breakthrough sequence. Missing viral load values were multiply imputed and represent averages across 20 datasets; solid points are observed values and open points are averages across 20 imputations. The Pearson correlation (r) is shown for vaccine (red circles; dashed line) and placebo (green squares; solid line) groups. Fitted lines from linear regression models are overlaid. (EPS) [file pone.0043396.s009.tif]

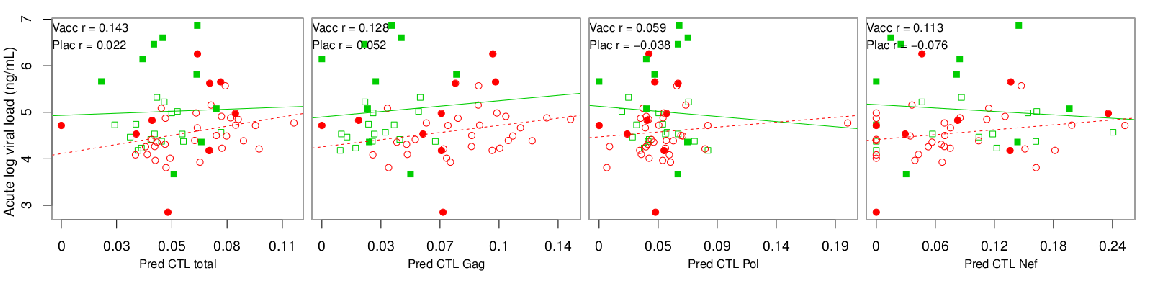

Supplement: Figure S10 — Association Between Acute Log10 Viral Load and Predicted CTL Epitope NetMHC Distance. The predicted CTL epitope distance between a breakthrough sequence and the MRKAd5 insert is the HIV-specific evolutionary (PAM) distance in peptides predicted to be epitopes in both sequences (based on NetMHC), averaged over a subject’s breakthrough sequences. Missing viral load values were multiply imputed and represent averages across 20 datasets; solid points are observed values and open points are averages across 20 imputations. The Pearson correlation (r) is shown for vaccine (red circles; dashed line) and placebo (green squares; solid line) groups. Fitted lines from linear regression models are overlaid. (EPS) [file pone.0043396.s010.tif]

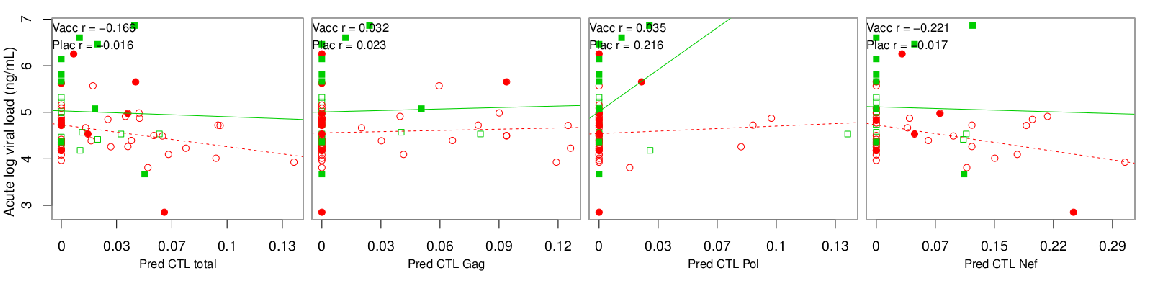

Supplement: Figure S11 — Association Between Acute Log10 Viral Load and Predicted CTL Epitope Epipred Distance. The predicted CTL epitope distance between a breakthrough sequence and the MRKAd5 insert is the HIV-specific evolutionary (PAM) distance in peptides predicted to be epitopes in both sequences (based on Epipred), averaged over a subject’s breakthrough sequences. Missing viral load values were multiply imputed and represent averages across 20 datasets; solid points are observed values and open points are averages across 20 imputations. The Pearson correlation (r) is shown for vaccine (red circles; dashed line) and placebo (green squares; solid line) groups. Fitted lines from linear regression models are overlaid. (EPS) [file pone.0043396.s011.tif]
