# Supplementary material for: MRKAd5 HIV-1 Gag/Pol/Nef Vaccine-Induced T-Cell Responses Inadequately Predict Distance of Breakthrough HIV-1 Sequences to the Vaccine or Viral Load
Source: PLoS One. 2012 Aug 27;7(8):e43396. doi: 10.1371/journal.pone.0043396 (PMC3428369; doi:10.1371/journal.pone.0043396)
Supplement: Table S1 — List of the 32 peptides covering the signature sites that were assessed using IFNγ ELISpot. (DOCX) [file pone.0043396.s012.docx]

**Table S1** List of the 32 peptides covering the signature sites that were assessed using IFNγ ELISpot

| **Signature site** | **Peptide** | **HXB2**  **position** |
| --- | --- | --- |
| Gag 84 | EELRSLYNTVA**T**LYC | 73-87 |
|  | SLYNTVA**T**LYCVHQK | 77-91 |
|  | TVA**T**LYCVHQKIDVK | 81-95 |
| Gag 211 | AMQMLKETINEEAA**E** | 197-211 |
|  | LKETINEEAA**E**WDRL | 201-215 |
|  | INEEAA**E**WDRLHPVH | 205-219 |
|  | AA**E**WDRLHPVHAGPI | 209-223 |
| Gag 403 | TVKCFNCGKVGHIA**K** | 389-403 |
|  | FNCGKVGHIA**K**NCRA | 393-407 |
|  | KVGHIA**K**NCRAPRKK | 397-411 |
|  | IA**K**NCRAPRKKGCWK | 401-415 |
| Pol 541 | ITTESIVIWGK**T**PKF | 530-544 |
|  | SIVIWGK**T**PKFKLPI | 534-548 |
|  | WGK**T**PKFKLPIQKET | 538-552 |
| Pol 721 | GIRKVLFLDGI**D**KAQ | 710-724 |
|  | VLFLDGI**D**KAQDEHE | 714-728 |
|  | DGI**D**KAQDEHEKYHS | 718-732 |
| Nef 64a | TNADCAWLEAQEDEE | 51-65 |
|  | CAWLEAQEDEEVGFP | 55-69 |
|  | EAQEDEEVGFPVRPQ | 59-73 |
|  | DEEVGFPVRPQVPLR | 63-77 |
| Nef 82 | RPQVPLRPMTY**K**GAV | 71-85 |
|  | PLRPMTY**K**GAVDLSH | 75-89 |
|  | MTY**K**GAVDLSHFLKE | 79-93 |
| Nef 116 | SQKRQDILDLWVY**H**T | 103-117 |
|  | QDILDLWVY**H**TQGYF | 107-121 |
|  | DLWVY**H**TQGYFPDWQ | 111-125 |
|  | Y**H**TQGYFPDWQNYTP | 115-129 |
| Nef 173 | GENNCLLHPMSQHG**I** | 159-173 |
|  | CLLHPMSQHG**I**EDPE | 163-177 |
|  | PMSQHG**I**EDPEKEVL | 167-181 |
|  | HG**I**EDPEKEVLEWRF | 171-185 |
