# Supplementary material for: MRKAd5 HIV-1 Gag/Pol/Nef Vaccine-Induced T-Cell Responses Inadequately Predict Distance of Breakthrough HIV-1 Sequences to the Vaccine or Viral Load
Source: PLoS One. 2012 Aug 27;7(8):e43396. doi: 10.1371/journal.pone.0043396 (PMC3428369; doi:10.1371/journal.pone.0043396)
Supplement: Materials S1 — (DOCX) [file pone.0043396.s013.docx]

**Supplementary Materials Text**

**1. Additional analyses of vaccine-induced anamnestic responses**

Depth of the post-infection T-cell response was compared between vaccine and placebo groups. Depth was defined at an amino acid site as the number of simultaneously elicited variant PTE-G peptides that cover the site, using HXB2 numbering. The depth distribution was compared between treatment groups using a Poisson generalized estimation equation model with exchangeable working correlation structure, conditioning on sites with 1 or more PTE-G responses. There was some evidence of less depth for vaccine recipients compared to placebo recipients, aggregating across Gag, Pol, and Nef (p = 0·05; Figure S2). The observations become sparse at the individual protein level, but there is some suggestion that this overall effect was driven by Nef and Pol, and that there was more depth among vaccine recipients in Gag.

To determine whether anamnestic responses could be traced to the signature sites, post-infection responses were measured using IFNγ ELISpot after stimulating with a set of 32 peptides covering 9 of the 10 signature sites (excluding Gag-475; see Table S1). Responses to these peptides were available for 71 of the 91 subjects; 18 had insufficient cells for testing and 2 had high background. The distribution of number of positive peptide responses, among those covering the signature sites, was compared between vaccine and placebo recipients using a Wilcoxon rank sum test, with the result that there was no evidence of a difference between treatment groups (p = 0·71; Figure S3).

The magnitude of the pre- and post-infection T-cell ICS responses were compared using the data for the 23 vaccine recipients whose pre-infection responses were tested post infection. Figure S4 shows the difference in log_10_ magnitude positive responses post- minus pre-infection. For each protein, a linear generalized estimating equations model was used to compare the mean difference to zero. There was evidence that on average post-infection magnitudes were higher than pre-infection in Gag (p = 0·01), but magnitudes were similar pre- and post-infection in Pol and Nef (p = 0·63 and 0·65, respectively).

**2. T-cell response based sieve analysis**

The T-cell response based sieve analysis assessed whether, within the measured pre-infection reactive epitopes, there was an unusually high rate of mismatches in vaccine recipient founder sequence epitopes compared to the corresponding insert epitopes. Because the primary analysis included overlapping reactive 15-mers within which there was likely only one reactive epitope, the analysis was repeated in two ways only counting one reactive 15-mer per vaccine recipient. The first selected the “immunodominant" reactive 15-mer as the one with the highest magnitude response while the second selected the most “conserved" reactive 15-mer [1] as the epitope with the lowest entropy. Using sequence data from placebo recipients, we computed the entropy of each epitope by averaging the entropy at each site within that epitope. To account for multiple sequences per subject we repeated the process 1,000 times based on data-sets formed by randomly sampling one sequence per subject. For a given set of epitopes, the one that most frequently had the lowest entropy was defined as the most conserved epitope.

Results are displayed in Figure S5. Considering all reactive 15-mers, there were more mismatches than expected by chance in Nef (p = 0·04), a non-significant trend for Pol (p = 0·13), and no significant evidence for Gag (p = 0·89). The average percent mismatch for vaccine recipients, with the median of the null distribution in parentheses, was calculated as 26.1% (23.3%) for Nef, 6.7% (5.5%) for Pol, and 8.5% (8.3%) for Gag. Using the most immodominant 15-mers, the analogous results were: 29.8% (26.6%) for Nef (p = 0.08), 7.5% (6.3%) for Pol (p = 0.33), and 8.5% (8.0%) for Gag (p = 0.81). Considering the most conserved 15-mers, the results were: 19.7% (17.2%) for Nef (p = 0.14), 4.2% (2.5%) for Pol (p = 0.09), and 5.9% (5.4%) for Gag (p = 0.71).

**3. Additional analyses of acute viral load**

Two additional analyses of acute viral load were conducted, in addition to the multiple imputation approach described in the main text. Each analysis relies on a different set of assumptions regarding the missing data mechanism, and therefore consistency in the results across methods is somewhat reassuring. An inverse-probability-weighted (IPW) analysis was performed in which the probability of having a missing viral load measure was estimated using a logistic regression model including HLA type, study week of diagnosis, number of weeks between the diagnosis visit and the previous visit, treatment assignment, and HSV-2 serostatus as predictors. The result was an estimated mean acute log viral load of 4·7 in vaccine recipients and 5·1 in placebo recipients (p = 0·58 t-test). A “complete case” analysis was also conducted including only the 27 subjects with observed acute viral load. This analysis is valid under the strong assumption that there are no measured or unmeasured variables that predict whether viral load was missing. The complete case analysis produced somewhat stronger evidence of a difference between treatment groups. The estimated mean acute log viral load was 4·9 in vaccine recipients and 5·5 in placebo recipients (p = 0·09 Wilcoxon rank sum; p = 0·22 t-test).

Figure S6 shows the distribution of acute viral load by treatment assignment and HLA group. The data were too sparse to perform formal statistical tests comparing vaccine and placebo recipients stratified by HLA group, but descriptively the average log acute viral load was lower in vaccine recipients compared to placebo recipients in the neutral and protective HLA groups.

**4. Association between acute viral load and epitope-based distance measures**

Figures S7-S11 show the association between acute viral load and each summary distance measure, for Gag, Pol, and Nef in total, and for each protein separately. Inferential results for these data are reported in the main text of the manuscript.

**5. Association between individual amino acid mutations and acute viral load in the vaccine group**

An analysis was performed to discover individual amino acid mutations associated with acute viral load using data for vaccine recipients only (n = 39). There was insufficient data to perform the analysis in placebo recipients. The analysis scanned through the Gag, Pol, and Nef (majority consensus) proteins to discover amino acid mismatches at sites that predict acute viral load. The statistical approach combined EEBoost with a semi-parametric efficient augmented IPW approach to estimate the mean log acute viral load as a function of the indicator variables for each amino acid mismatch [2]. Missing viral load values were imputed as described in the methods section of the main text and the probability of having a missing viral load measurement was estimated as described in Supplementary Materials Section 3. The statistical significance of the mismatches was assessed using the two-stage permutation procedure described in Gilbert *et al.* [3] which controls the expected number of false-positive findings (amino acid positions) at one for each protein.

The results were as follows. One site in Pol and 1 site in Nef were identified as being associated with acute viral load. Given that the analysis controlled the expected number of false positives at one per protein, these are likely false positive discoveries. Five amino acid mismatches were identified in Gag. Interestingly, 3 of the 5 sites in Gag are located within 9 amino acids of a signature site and 2 are covered by 15-mers which generated pre-infection T-cell responses (see Figure 4 of the main text). Mismatched amino acid at sites D93E and E460A were associated with 0·12 and 0·30 higher average log viral load than matched amino acid. This suggests that T-cells, induced by vaccination and/or by HIV infection, may have recognized the epitope covering these sites and were able to suppress viral replication. However neither of these sites was detected in the analysis which compared acute viral load between vaccine and placebo recipients with an insert-matched amino acid at each individual site (see main text Results). Mismatched amino acid at sites Q127K, T204V, and E482D were associated with 0.34, 0.25, and 0.39 lower average log viral load than matched amino acid. This suggests a potential fitness cost associated with these mutations.

**References**

1 Barouch DH, O'Brien KL, Simmons NL *et al.* Mosaic HIV-1 vaccines expand the breadth and depth of cellular immune responses in rhesus monkeys. *Nat Med.* 2010; **16**: 319–23.

2 Wolfson J. EE boost: A general method for prediction and variable selection based on estimating equations. *J Am Stat Assoc* 2011; **106**: 296-305.

3 Gilbert PB, Wu C, Jobes DV. Genome scanning tests for comparing amino acid sequences between groups. *Biometrics* 2008; **64**: 198–207.
